# Editorial: Computational protein function prediction based on sequence and/or structural data

**DOI:** 10.3389/fbinf.2025.1705252

**Published:** 2025-10-31

**Authors:** Yaan J. Jang

**Affiliations:** Department of Biochemistry, University of Oxford, Oxford, United Kingdom

**Keywords:** protein function prediction, machine learning, deep learning, protein sequence, protein structure

In recent years, the integration of computational and machine learning techniques has dramatically advanced our understanding of protein function and engineering. As proteins underpin virtually every biological process, accurate predictions of their functions—whether based on sequence or structural data—are critical to unraveling their roles in health, disease, and therapeutics. This editorial explores how cutting-edge computational strategies are shaping the future of protein sciences, as illustrated by the contributions in this Research Topic. By embracing both evolutionary data and state-of-the-art technologies like deep learning and molecular dynamics simulations, these studies illuminate new pathways for advancing drug development, vaccine design, and pathogen-interaction research.

At the heart of protein function prediction is the challenging task of deciphering the link between protein sequences, their three-dimensional structures, and the biological functions they support. While protein sequences provide a genetic blueprint, their true functional capabilities are often embedded in the spatial arrangement of amino acids and the resulting folding patterns. Recent breakthroughs in computational biology have allowed researchers to use sequence data and structural features to predict protein functions with unprecedented accuracy (Gligorijević et al., 2021; Jang et al., 2024).

Machine learning and deep learning approaches, such as DeepPredict (Alanazi et al.) and DCMA (Zhang et al.), are enhancing the landscape of protein function prediction. These algorithms leverage large datasets to identify subtle patterns in sequence-structure-function relationships, enabling the prediction of secondary structures, solvent accessibility, and backbone dihedral angles with improved precision. Importantly, these methods do not only enhance prediction accuracy; they also reduce computational demands, enabling large-scale analysis that was previously unfeasible. Several studies in this Research Topic highlight the utility of such AI-based tools for practical biomedical applications, from antigen design to drug discovery.

One of the most compelling uses of these predictive methods is in the realm of vaccine development. For instance, AI-guided approaches have revolutionized the design of broadly protective antigens by predicting how viral proteins interact with immune receptors (Motiwala et al.; Chesterman et al.). These insights are not just theoretical—they are directly applicable to real-world challenges like the development of vaccines against evolving pathogens. This Research Topic includes contributions that exemplify how computational tools can accelerate vaccine design by identifying critical epitopes and optimizing immune response profiles. In particular, the studies focusing on viral infections, including the investigation of SARS-CoV-2 cytokine interactions and the engineering of antiviral peptides, underscore the role of computational methods in addressing urgent global health threats. One such example is the design of cross-reactive antigens using machine learning, where computational models were employed to predict fHbp properties and design mutants that could potentially be used in next-generation vaccine platforms (Chesterman et al.).

In addition to antigen design, another area where protein function prediction plays a pivotal role is in drug discovery. The ability to predict protein-ligand and protein-protein binding sites has far-reaching implications for designing therapeutic interventions. Many contributors to this Research Topic explore novel techniques for predicting these binding sites, with a particular emphasis on the challenges of pathogen-host interactions. By studying protein-protein interactions in pathogens such as *Enterobacter cloacae* (Motiwala et al.), or host-virus interactions like those between the human immune system and SARS-CoV-2, these studies are advancing our understanding of the molecular underpinnings of disease. By accurately predicting how proteins interact, researchers can identify new drug targets and design more effective treatments. Notably, large-scale molecular docking and PhIP-Seq (Huang et al.) approaches are being employed to uncover complex host-virus interactions, aiding in the discovery of novel therapeutic strategies.

Moreover, this Research Topic highlights the growing importance of high-throughput techniques and large-scale molecular simulations in structural biology. Tools such as PhIP-Seq (Huang et al.), which enables comprehensive antibody profiling, and large-scale molecular docking, which uncovers complex host-virus interactions, are helping to unravel the intricate web of protein interactions that govern biological processes. These approaches are essential for mapping the full spectrum of pathogen-host dynamics and for designing strategies that can mitigate the impact of infectious diseases. Additionally, a computational framework for virtual screening of cytokine and coronavirus nucleocapsid protein interactivity has accelerated the discovery of effective interventions against viral threats, showcasing the complementary nature of deep learning-based models and semi-physicochemical methods (Tomezsko et al.).

Underlying all these efforts is an appreciation for the evolutionary data that drives protein function. Understanding how proteins evolve—and how evolutionary pressures shape their structure and function—is a key aspect of protein engineering. In this context, evolutionary algorithms and molecular dynamics simulations have emerged as invaluable tools. By simulating how proteins have adapted over time and predicting how they might evolve in the future, these methods provide critical insights into how we can design proteins with desired functions. Whether it’s the development of enzymes with novel catalytic properties or the engineering of proteins with enhanced binding affinities, evolutionary data are integral to rational protein design. The study of Nipah virus (NiV) and its interaction with host receptors highlights the importance of understanding viral evolution and using computational approaches to develop novel antiviral peptides for therapeutic applications (Sajjad et al.).

The diversity of studies presented here emphasizes the breadth of protein function prediction and engineering, ranging from the design of therapeutic antibodies to the prediction of protein-protein interactions and the identification of antiviral peptides. Collectively, these contributions highlight the transformative power of computational methods in modern biomedical research. They also reinforce the growing recognition that understanding protein function requires an integrated approach, combining sequence data, structural insights, and evolutionary analysis.

Looking ahead, the convergence of deep learning, molecular dynamics, and high-throughput techniques promises to further enhance our ability to predict and engineer protein functions with ever greater accuracy and efficiency. As these computational strategies continue to evolve, they will likely open new frontiers in protein science, providing invaluable tools for tackling complex challenges in drug discovery, vaccine development, and beyond.

In conclusion, this Research Topic of articles provides a timely and comprehensive overview of the current state of computational protein function prediction, showcasing how innovative technologies are driving progress in both fundamental and applied protein research. The future of protein engineering lies in our ability to leverage these advances to design proteins with specific functions, offering exciting possibilities for the development of novel therapeutics and biotechnologies.
